# Supplementary material for: Evaluating and Using Observational Evidence: The Contrasting Views of Policy Makers and Epidemiologists
Source: Front Public Health. 2016 Dec 6;4:267. doi: 10.3389/fpubh.2016.00267 (PMC5138237; doi:10.3389/fpubh.2016.00267)
Supplement: Supplementary file 1 [file Data_Sheet_1.DOCX]

**Epidemiology interview questions:**

1. In your opinion, what constitutes high quality observational research?
2. What is your view on the typical hierarchy of evidence and how research evidence is rated?
3. Do you know anything about current rating systems for grading evidence? If yes, which systems have you heard of, and where are they used? AND, do you use a specific rating system to evaluate research evidence in your own practice? If yes, what is it? If no, how do you evaluate the quality of research evidence?
4. In your view what are the consequences/costs of inappropriate rating of observational research? Is it a concern and why?

**Policymaker web survey questions**

1. As far as your concerned, how significant are the barriers listed below to knowledge translation from research into policy in your current work environment?
2. In your view knowledge translation is the responsibility of:
   1. Knowledge producers (e.g. researchers, research institutions)
   2. Knowledge users (e.g. policy makers, clinicians)
   3. Knowledge translations professionals (e.g. knowledge brokers)
   4. A combination of the above/other
3. If you could ask researchers to undertake a study on an issue relevant to your area of work, what sort of study would you prefer and what would you want to find out?
4. Have you used any ACT Health reports in formulating new policies? If so, which one(s)?
5. In your opinion what constitutes high quality evidence?
6. Please give each of these types of research a “quality of evidence” rating, according to your understanding of quality evidence. Indicate your preferred research method9s0 when building an evidence base.
   1. RCT
   2. Systematic reviews
   3. Observational research
   4. Meta-analyses
   5. Expert opinion (e.g. clinician)
   6. Cohort studies
   7. Case reports/case studies
   8. Case-series
   9. Government reports
   10. Qualitative research
   11. other
7. Please indicate how important each of the following are when you evaluate evidence that might contribute to policy:
   1. Reputation of journal
   2. Reputation of researcher or clinician
   3. Consistency and strength of evidence
   4. Type of evidence (e.g. observational study vs. RCT)
   5. Recency of evidence
   6. Evidence that is locally applicable
   7. Quality of the data (systematic review or large study vs small poorly designed)
   8. Risk of bias in the evidence
   9. The evidence ‘backs up’ the policy
8. When working on a policy development task: please indicate how often you typically utilise or consult the following sources of evidence.
   1. Expert opinion (e.g. clinicians)
   2. ACT Health statistical data
   3. External statistical data
   4. Existing academic research
   5. Publications from trusted organisations
   6. Guidelines
   7. Similar policy experiences from other jurisdictions
   8. Academics
   9. Consultant organisations
   10. Consumer views
9. When working on a policy development task: please indicate how easy/difficult you typically find it to understand evidence from the following sources. (as above)

**Policymaker interviews questions:**

1. Could you walk me through a recent occasion when you were asked to contribute regarding a policy decision in an area that required the input of research evidence?
2. What are your preferred sources of evidence? Are the same sources the most useful and/or influential in policy discussions- if not which ones are?
3. In your opinion, what does “high quality evidence” mean? What sources would you include under this definition and what provisos would you put on them?
4. What are the biggest obstacles or barriers when it comes to accessing and using research?
5. To what extent do you think research influences policy outcomes?
6. What factors do you think would make research more useful and/or accessible for policy makers?
7. What factors do you believe could make policy-makers more receptive to research?
