# Supplementary material for: Evaluating and Using Observational Evidence: The Contrasting Views of Policy Makers and Epidemiologists
Source: Front Public Health. 2016 Dec 6;4:267. doi: 10.3389/fpubh.2016.00267 (PMC5138237; doi:10.3389/fpubh.2016.00267)
Supplement: Supplementary file 2 [file Data_Sheet_2.PDF]

Evidence Grading Instruments

Broadly speaking, grading instruments are a means of quantifying the quality of research evidence. They usually do this through the provision of a rating score or 'grade' that allows the user to compare the quality of different pieces of research. These instruments provide criteria for deriving these grades, as well as some form of instruction as to how to apply those criteria. Some of these instruments are described by their creators as being appropriate for the grading of multiple kinds of research, while others are specialised for use in evaluating only specific research designs (e.g. randomised control trials, observational studies, or qualitative studies). Additionally, instruments can be divided into those that are designed for the rating of single pieces of evidence (i.e. an individual research paper) and those that are designed to evaluate a body of evidence (such as a systematic review or a meta-analysis).

Examples

GRADE

The most widely used grading systems is GRADE (Grading of Recommendations, Assessment, Development and Evaluations). This system is used by the Agency for Healthcare Research and Quality (AHRQ-USA), American College of Physicians (USA), British Medical Journal (BMJ), BMJ Clinical Evidence, COMPUS at The Canadian Agency for Drugs and Technologies in Health, The Cochrane Collaboration, National Institute for Health and Clinical Excellence (NICE - UK), and the World Health Organization (WHO) – and by many more institutions. GRADE guides the user into deriving a grade for both the quality of evidence, and the strength of recommendation. Information on GRADE can be found at http://www.gradeworkinggroup.org/.

NHMRC - FORM

In Australia the National Health and Medical Research Council (NHMRC) uses grading criteria known as FORM. Consisting of five components (evidence base, consistency, clinical impact, generalisability and applicability), the FORM framework is used by clinical guideline developers to derive a strength of recommendation and to consider implementation implications for each recommendation. More information can be found at: http://www.biomedcentral.com/1471-2288/11/23.

OTHER GRADING INSTRUMENTS

CASP: Details available at the Critical Appraisal Skills Program website: http://www.casp-uk.net/

Downs & Black Checklist: Downs, S. H., & Black, N. (1998). The feasibility of creating a checklist for the assessment of the methodological quality both of randomised and non-randomised studies of health care interventions. *Journal of Epidemiology and Community Health*, *52*(6), 377-384.

GATE: Jackson, R., Ameratunga, S., Broad, J., Connor, J., Lethaby, A., Robb, G., ... & Heneghan, C. (2006). The GATE frame: critical appraisal with pictures. *Evidence Based Medicine*, *11*(2), 35-38

JADAD: Jadad, A. R., Moore, R. A., Carroll, D., Jenkinson, C., Reynolds, D. J. M., Gavaghan, D. J., & McQuay, H. J. (1996). Assessing the quality of reports of randomized clinical trials: is blinding necessary? *Controlled clinical trials*, *17*(1), 1-12.

NICE: Details available at National Institute for Health and Clinical Effectiveness website: http://www.nice.org.uk:80/aboutnice/howwework/developingniceclinicalguidelines/clinicalguidelinedevelopmentmethods/clinical_guideline_development_methods.jsp

NOS: Wells, G. A., Shea, B., O'Connell, D., Peterson, J., Welch, V., Losos, M., & Tugwell, P. (2000). The Newcastle-Ottawa Scale (NOS) for assessing the quality of nonrandomised studies in meta-analyses. Details available at: http://www.medicine.mcgill.ca/rtamblyn/Readings/The%20Newcastle%20-%20Scale%20for%20assessing%20the%20quality%20of%20nonrandomised%20studies%20in%20meta-analyses.pdf

NSF-LTC: Turner-Stokes, L., Harding, R., Sergeant, J., Lupton, C., & McPherson, K. (2006). Generating the evidence base for the National Service Framework for Long Term Conditions: a new research typology. *Clinical medicine*, *6*(1), 91-97.

RoBANS: Park, J., Lee, Y., Seo, H., Jang, B., Son, H., & Kim, S. Y. (2011). Risk of bias assessment tool for non-randomized studies (RoBANS): development and validation of a new instrument. In *Proceedings of the 19th Cochrane Colloquium* (pp. 19-22).

SIGN: Scottish Intercollegiate Guidelines Network methodology available at: http://www.sign.ac.uk/guidelines/fulltext/50/index.html

SORT: Ebell, M.H., Siwek, J., Weiss, B.D., Woolf, S.H., et al. (2004). Strength of Recommendation Taxonomy (SORT): a patient-centered approach to grading evidence in the medical literature. *American Family Physician, 69,* 549-57.
